# Influence of timing of Levosimendan administration on outcomes in cardiac surgery

**DOI:** 10.3389/fcvm.2023.1213696

**Published:** 2023-07-26

**Authors:** Fridtjof Schiefenhövel, Christian Berger, Liubov Penkova, Herko Grubitzsch, Bernhard Haller, Alexander Meyer, Matthias Heringlake, Michael Sander, Joachim M. Erb, Felix Balzer, Sascha Treskatsch

**Affiliations:** ^1^ Department of Anaesthesiology and Intensive Care (AINS), Medical Center Rechts der Isar, School of Medicine, Technical University of Munich, Munich, Germany; ^2^ Institute for Artificial Intelligence and Informatics in Medicine (AIIM), Chair of Medical Informatics, Medical Center Rechts der Isar, School of Medicine, Technical University of Munich, Munich, Germany; ^3^ Charité—Universitätsmedizin Berlin, Corporate Member of Freie Universität Berlin and Humboldt Universität zu Berlin, Institute of Medical Informatics, Berlin, Germany; ^4^ Department of Anaesthesiology and Intensive Care Medicine, Charité Campus Benjamin Franklin, Charité—Universitätsmedizin Berlin, Corporate Member of Freie Universität and Humboldt Universität zu Berlin, Berlin, Germany; ^5^ Department of Anaesthesiology and Intensive Care Medicine, Charité Campus Mitte and Charité Campus Virchow, Charité—Universitätsmedizin Berlin, Corporate Member of Freie Universität and Humboldt Universität zu Berlin, Berlin, Germany; ^6^ Klinik für Kardiovaskuläre Chirurgie, Campus Virchow Klinikum, Charité—Universitätsmedizin Berlin, Corporate Member of Freie Universität and Humboldt Universität zu Berlin, Berlin, Germany; ^7^ Department of Cardiothoracic and Vascular Surgery, German Heart Center Berlin, Berlin, Germany; ^8^ Department of Anaesthesia, Heart and Diabetes Center, Klinikum Karlsburg, Karlsburg, Germany; ^9^ Department of Anaesthesiology, Intensive Care Medicine and Pain Therapy, University Hospital Gießen UKGM, Justus-Liebig University Gießen, Gießen, Germany; ^10^ Clinic for Anaesthesiology, Intermediate Care, Prehospital Emergency Medicine and Pain Therapy, University Hospital Basel, Basel, Switzerland

**Keywords:** Levosimendan, cardiac surgery, high-risk patients, low cardiac output syndrome, mortality, outcome

## Abstract

**Purpose:**

Though a subgroup analysis has shown improved survival for patients suffering severely reduced ventricular function undergoing coronary artery bypass grafting, RCTs were not able to demonstrate overall beneficial effects of perioperative Levosimendan in cardiac surgery. This might be due to Levosimendan’s pharmacokinetics reaching a steady-state concentration only 4–8 h after administration. Thus, this study now analysed the influence of timing of Levosimendan administration on perioperative outcome in cardiac surgery patients preoperatively presenting with severely reduced ventricular function and therefore considered at high-risk for intra- or postoperative low cardiac output syndrome. We hypothesized that prolonged preoperative Levosimendan administration (“preconditioning”) would reduce mortality.

**Methods:**

All adult patients undergoing cardiac surgery between 2006 and 2018 perioperatively receiving Levosimendan were included in this retrospective, observational cohort study (*n* = 498). Patients were stratified into 3 groups: Levosimendan started on the day prior to surgery (“preop”), Levosimendan started on the day of surgery (“intraop”) or post ICU admission (“postop”). After propensity score matching (PSM) was performed, outcomes defined according to proposed standard definitions for perioperative outcome research were compared between groups.

**Results:**

After PSM, there were no significant differences in patients’ characteristics, comorbidities and type/priority of surgery between groups. Compared to intraop or postop Levosimendan treatment, preop treated patients had significantly lower in-hospital-mortality (preop vs. intraop. vs. postop = 16,7% vs. 33,3% vs. 42,3%), duration of mechanical ventilation and rate of continuous renal replacement therapy.

**Conclusions:**

Prolonged preoperative treatment with Levosimendan of cardiac surgery patients preoperatively presenting with severely reduced left ventricular function might be beneficial in terms of postoperative outcome. Our results are in line with recent experts’ recommendations concerning the prolonged perioperative use of Levosimendan. We strongly recommend that future randomized trials include this “preconditioning” treatment as an experimental arm.

## Introduction

Levosimendan is a calcium-sensitising inotropic drug, which increases cardiac contractility and reduces cardiac afterload without significantly increasing myocardial oxygen consumption (1). Several trials in patients suffering from acute heart failure have shown benefits of Levosimendan treatment (2–6), although one such trial failed to show benefits (2–7). The perioperative use of Levosimendan in cardiac surgery and its effect on outcome of patients with preoperatively reduced left ventricular function has been the subject of several large randomized controlled trials (RCT) (8–10). While the results of these have not shown a clear benefit across the whole range of cardiac surgery patients, subgroup analysis has shown a significantly improved survival for patients suffering from coronary artery disease and severely reduced ventricular function undergoing coronary artery bypass grafting (CABG) (8, 11). Furthermore, another prospective study comparing an historical cohort with a prospective one (12) and one randomized trial (13) have shown benefits of prolonged preoperative treatment with Levosimendan (“preconditioning”) in patients with moderate to severe left ventricular dysfunction undergoing elective coronary artery bypass surgery.

One possible reason why the abovementioned RCTs have failed to show a clear benefit of perioperative Levosimendan might be its peculiar pharmacokinetics. The steady-state concentration of Levosimendan is only reached after 4–8 h, and its active metabolite, first detectable 12 h after administration, peaks at 48–78 h after the beginning of administration (14, 15). We thus hypothesize that the optimal perioperative administration of Levosimendan might have to start well before cardiac surgery, e.g., 12–24 h prior. In a previous retrospective study, early administration of Levosimendan, i.e., start of administration following the induction of anaesthesia or start of administration intraoperatively, was associated with increased survival in contrast to a late administration postoperatively on intensive care unit (ICU) (16).

With respect to these data, the standard operating procedure (SOP) concerning the perioperative use of Levosimendan has been changed at a tertiary hospital in 2013 towards a strong recommendation to preoperatively screen all patients undergoing cardiac surgery for severely reduced ventricular function and to precondition such patients regarded as being at high-risk for developing low cardiac output syndrome (LCOS) with Levosimendan one day prior to surgery. In this retrospective single-centre cohort study, we have now analysed effects of timing of Levosimendan administration on perioperative outcome in a large cohort of cardiac surgery patients. As primary objective, we hypothesized that prolonged preoperative (12–16 h) Levosimendan treatment as a routine clinical practice in a heterogeneous cohort of cardiac surgery patients exhibiting preoperatively severely reduced left ventricular function considered high-risk reduces mortality. As secondary objective, we aimed to determine if potential outcome effects of Levosimendan differed between isolated CABG and other cardiac surgical procedures like isolated valve or combined CABG and valve surgery.

## Materials and methods

### Design and inclusion criteria

After approval of the Charité Ethics Committee, Berlin, Germany (study ID no.: EA4/239/19), we reviewed charts and data derived from 2 electronic patient data management systems (COPRA System GmbH, Sasbachwalden, Germany, and SAP AG, Walldorf, Germany). The requirement for informed consent from the study subjects was waived by the Ethics Committee due to the retrospective nature of the study. This observational cohort study was performed in accordance with the relevant guidelines and regulations and based on previously published approaches and in accordance with the Strengthening the Reporting of Observational Studies in Epidemiology statement (STROBE) (17–19). All patients admitted to our intensive care units between 2006 and 2018 scheduled for or after on-pump cardiac surgery identified by German OPS codes (5–35, 5–36; excluding 5–35A, i.e., minimally invasive valve replacement) that were treated with Levosimendan were eligible for inclusion in the study. Perioperative clinical data were extracted from the two digital/electronic patient data management systems and inserted into an anonymized study database. Patients under the age of 18 at the time of surgery were excluded.

### Perioperative management

Cardiac surgery, anaesthesia and hemodynamic management were performed in accordance with the department’s SOPs displaying the most recent recommendations at that respective time (20). Normothermic cardiopulmonary bypass (CPB) was established with a flow of 2.5 L/min/m^2^ and an arterial pressure ≥60 mmHg. Cardioplegic arrest was induced and maintained by intermittent administration of antegrade warm potassium-enriched blood (21) or Bretschneider’s solution according to the surgeons preference.

Perioperative goal-oriented hemodynamic support was established according to institutional standards guided by the German S3 guidelines using echocardiography as the primary diagnostic/monitoring tool (20, 22). In case of difficult CPB weaning despite hemodynamic optimization, an Intra-Aortic Balloon Pump (IABP) and/or ventricular assist device (VAD) were placed intraoperatively according to the team’s assessment. After chest closure, all patients were transferred intubated and mechanically ventilated to the ICU aiming at a fast-track concept (e.g., extubation within 6 h when cardiopulmonary stable). In the following patients were then transferred to the IMCU and afterwards to the normal ward before hospital discharge.

### Standard operating procedure concerning Levosimendan

**Before 2013**, the respective SOP allowed the administration of Levosimendan based on an individualized approach. Additionally, elective cardiac patients were not actively screened preoperatively for underlying heart failure in order to routinely administer Levosimendan. Levosimendan was thus most often infused on the day of surgery or later at the discretion of the attending cardiac anaesthesiologist if severe impairment of left ventricular systolic function (LVEF ≤35%) and/or LCOS became clinically meaningful. Most importantly, Levosimendan was not administered within a prespecified pre-emptive clinical pathway.

**From 2013 onwards**, the revised SOP called for active preoperative screening of all cardiac surgery patients to identify those exhibiting severe impairment of left ventricular systolic function (LVEF ≤35%). Involving our colleagues from the department of cardiac surgery in a shared decision-making process, Levosimendan was then preoperatively administered in these patients. This was performed in an ICU/IMCU setting under invasive blood-pressure measurement. Typically, intravenous administration started in the early afternoon one day before surgery, resulting in 70%–80% of the loading dose of Levosimendan (12,5 mg, see below) having been administered before the induction of anaesthesia, with the remaining portion continuing to be administered afterwards. Levosimendan was always administered as a single continuous infusion (12.5 mg Levosimendan in 50 ml 5% Glucose) at a rate of 0.1 μg/kg per minute, and patients treated with Levosimendan did not receive phosphodiesterase-III inhibitors for at least 5 days.

For all other cardiac surgical patients, the recommendation concerning the administration of Levosimendan remained unchanged, i.e., allowing an individualized approach.

### Definition of groups

All digital records of patients undergoing major cardiac surgery between 2006 and 2018 were filtered for the administration of Levosimendan. We then stratified patients into three groups: Patients whose Levosimendan administration started on the day prior to surgery (“preop”), i.e., having been treated prolonged preoperatively. As mentioned before, this preconditioning was performed on ICU/IMCU, depending on capacity, under invasive blood-pressure measurement. Patients whose first administration of Levosimendan started on the day of surgery, i.e., in the operating theatre during surgery or the associated anaesthesiological procedures, were labelled “intraop”. Patients who received the first dose of the drug postoperatively on ICU were labelled “postop”. We included only those patients who received the first administration up to 36 h before the initial cardiac surgery or up to 120 h afterwards. See Consort flowchart (Supplementary Figure S3) for an overview.

### Outcome variables

Outcomes were defined according to proposed standard definitions for perioperative outcome research (23). ICU mortality was the study’s primary outcome. In-hospital mortality, length of stay in-hospital and ICU, duration of invasive mechanical ventilation, incidence of renal dysfunction defined by KDIGO stage greater than or equal to 1 (24), and the need of continuous renal replacement therapy (CRRT) excluding cases with pre-existing chronic renal insufficiency were chosen as secondary outcomes. We calculated continuous outcomes like length of stay and mechanical ventilation twice: once an aggregated value including all patients in this group, once excluding patients who had died and reporting the aggregated value only for survivors. The reason being that we consider it valuable to report continuous outcomes that have been corrected for biases, e.g., early deaths.

### Statistical analysis

Statistical analyses of the anonymized dataset were undertaken with a *p* value below 0.05 regarded as significant. Significance among groups was analysed by *t*-test or ANOVA in the case of continuous normal-distributed values, by the nonparametric Kruskal-Wallis test in the case of non-normal distributed values and by chi-squared or Fisher’s exact tests for qualitative data. Results were given as median and interquartile range in non-normal distributed values, otherwise mean ± standard deviation. Numbers with percentages characterize qualitative observations. All tests should be understood as constituting explorative analysis, as no adjustment for multiple testing has been made. Propensity score matching (PSM) was performed on the variables age, sex, type of surgery, surgical urgency, Charlson Comorbidity Index, congestive heart failure, NYHA greater or equal to 3, pulmonary hypertension, chronic obstructive pulmonary disease, arterial hypertension, peripheral arterial disease and chronic renal insufficiency. These variables were chosen because of their known impact on postoperative outcome. Matching method was “nearest neighbour”, ratio was 1, and caliper was set to 0.2; matching was done in two rounds, i.e., both the group of patients that was treated with Levosimendan intraoperatively and the group that was treated with Levosimendan postoperatively was matched to the group that was preconditioned with Levosimendan. (S)MDs were depicted graphically for all matchings performed in the supplemented loveplots. Statistical analyses were performed using the R Project of Statistical Computing 4.3.0 (25); additionally we used the packages cobalt 4.5.1 (26), compareGroups 4.7.0 (27), ggpubr 0.6.0 (26), MatchIt 4.5.3 (28), tableone 0.13.2 (29) and tidyverse 2.0.0 (30).

## Results

### Study cohort

Out of 11,198 patients that underwent on-pump cardiac surgery during the specified period, 498 received Levosimendan during their perioperative index stay within 36 h before the start of the operation and up to 120 h after the start of the operation (see Consort flowchart, Supplementary Figure S3).

### Timing of Levosimendan

**Figure 1:**
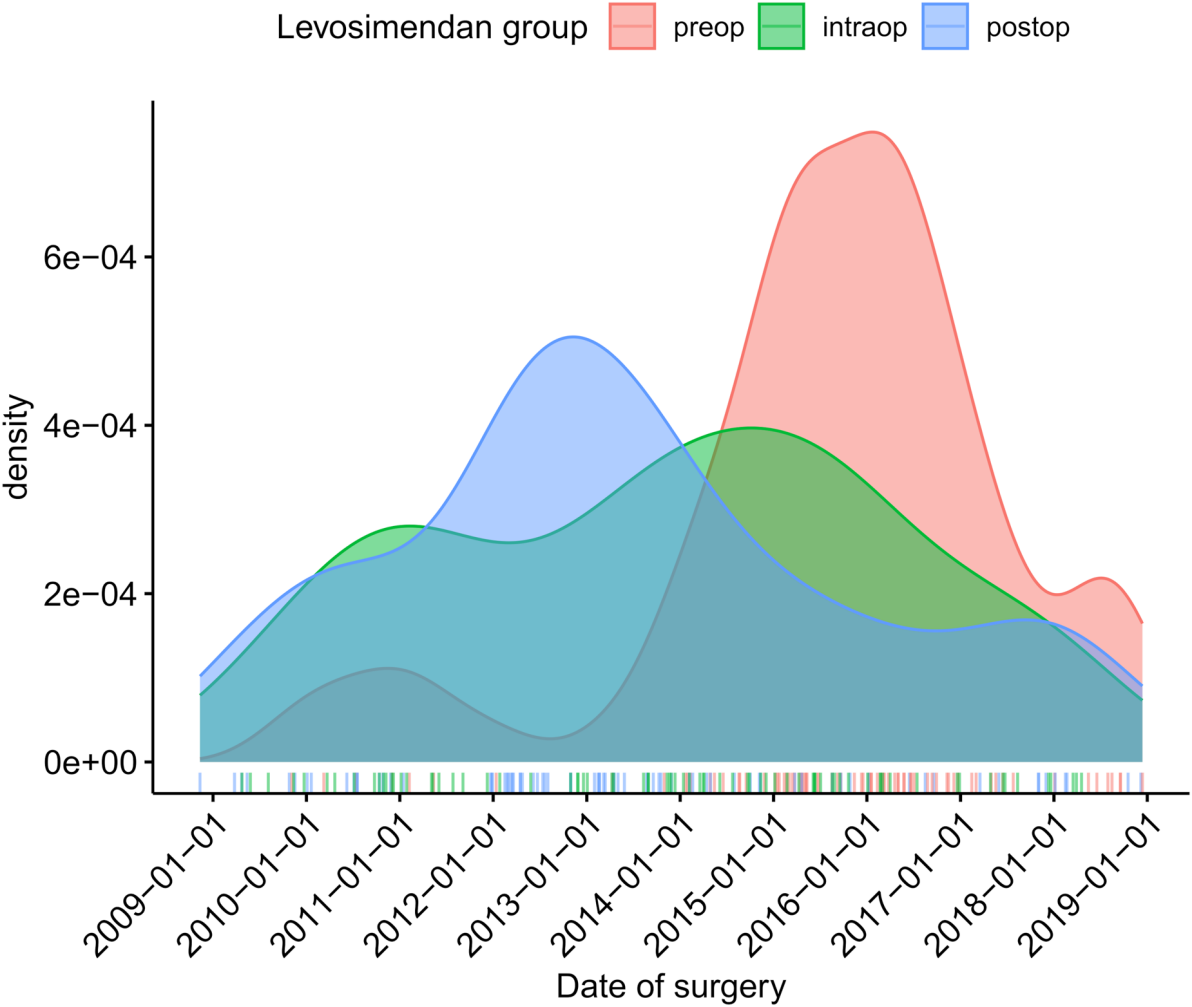
Timing of Levosimendan administration of matched population in normalized fractions (density plot).

The change in our hospital’s SOP led to a drastically altered timing of Levosimendan. As can be seen in Figure 1: the number of Levosimendan-preconditioned patients increased strongly in the years following 2013 (graph uses matched population). Please find detailed analysis of distribution of delays between start of Levosimendan treatment and start of operation in Supplementary Figure S1 (graph uses matched patients). See Supplementary Figure S2 for absolute numbers of unmatched population.

### Morphometry

Patients’ characteristics and outcome measures for the unmatched study population are presented in Supplementary Tables S1 and S2. Baseline characteristics of the resulting matched groups of patients that received Levosimendan within the specified time frame are shown in Table 1. After PS matching, there were no significant differences in age, sex, type of intervention, priority of surgery and selected pre-existing medical conditions between the different groups. For a graphical presentation of standardized mean differences of variables used for matching, see corresponding love plots in the supplements. The majority of patients was male and received elective CABG surgery. For subgroup analyses of matched patients who received either elective CABG or valve or combined surgery see Supplementary Tables S3–S6. For an analysis of a subset of patients, excluding patients who received Levosimendan intra- or postoperatively after January 2013, see Supplementary Tables S7 and S8.

**Table 1:** Baseline characteristics.

	[ALL]	Preop	Intraop	Postop	P. overall	*N*	MD
	*N = 234*	*N = 78*	*N = 78*	*N = 78*			
Age*	71.0 [62.0; 76.0]	71.0 [62.0; 75.0]	71.0 [63.0; 76.0]	69.5 [61.0; 76.0]	0.716	234	0.089
Sex*					0.543	234	0.117
M	197 (84.2%)	66 (84.6%)	63 (80.8%)	68 (87.2%)			
W	37 (15.8%)	12 (15.4%)	15 (19.2%)	10 (12.8%)			
BMI	27.1 [24.4; 30.3]	27.6 [23.6; 30.1]	26.9 [24.5; 30.3]	27.2 [24.9; 30.6]	0.965	195	0.049
Type of surgery*					0.986	234	0.064
CABG	155 (66.2%)	50 (64.1%)	52 (66.7%)	53 (67.9%)			
Combined	27 (11.5%)	10 (12.8%)	9 (11.5%)	8 (10.3%)			
Valve	52 (22.2%)	18 (23.1%)	17 (21.8%)	17 (21.8%)			
Urgency*					0.876	234	0.054
Elective	154 (65.8%)	53 (67.9%)	51 (65.4%)	50 (64.1%)			
Urgent/emergent	80 (34.2%)	25 (32.1%)	27 (34.6%)	28 (35.9%)			
CCI*	6.00 [5.00; 8.00]	6.50 [5.00; 8.00]	6.00 [5.00; 8.00]	6.00 [5.00; 7.00]	0.756	234	0.053
CHF*	217 (92.7%)	73 (93.6%)	71 (91.0%)	73 (93.6%)	0.776	234	0.064
NYHA ≥3*	203 (86.8%)	69 (88.5%)	66 (84.6%)	68 (87.2%)	0.771	234	0.075
PAH*	62 (26.5%)	19 (24.4%)	23 (29.5%)	20 (25.6%)	0.752	234	0.077
CAD	203 (86.8%)	66 (84.6%)	68 (87.2%)	69 (88.5%)	0.771	234	0.075
COPD*	43 (18.4%)	14 (17.9%)	14 (17.9%)	15 (19.2%)	0.972	234	0.022
AHTN*	176 (75.2%)	58 (74.4%)	61 (78.2%)	57 (73.1%)	0.742	234	0.080
PAD*	50 (21.4%)	16 (20.5%)	17 (21.8%)	17 (21.8%)	0.975	234	0.021
Diabetes	149 (63.7%)	55 (70.5%)	46 (59.0%)	48 (61.5%)	0.290	234	0.162
CRI*	77 (32.9%)	28 (35.9%)	24 (30.8%)	25 (32.1%)	0.778	234	0.073

Baseline characteristics of matched patients that received Levosimendan within the specified time frame.

* Matched on age + sex + type of surgery + surgical urgency + Charlson Comorbidity Index (CCI) + congestive heart failure (CHF) + NYHA ≥3 + pulmonary hypertension (PAH) + chronic obstructive pulmonary disease (COPD) + arterial hypertension (AHTN) + peripheral arterial disease (PAD) and chronic renal insufficiency (CRI). Groups: preop, Levosimendan started at least one day before surgery, intraop, L. started on the day of surgery, postop, L. started one day after surgery or later. CAD, coronary artery disease; COPD, chronic obstructive pulmonary disease; PAD, peripheral arterial disease; CRI, chronic renal insufficiency. MD, mean differences, standardized mean differences for continuous variables (Age, BMI, CCI).

### Outcome parameters

After matching, preconditioned patients had significantly lower ICU- and in-hospital-mortality, duration of mechanical ventilation and rate of continuous renal replacement therapy (CRRT) (Table 2) when compared to patients who received Levosimendan on the day of the surgery or later. Length of ICU stay, length of overall hospital stay and duration of mechanical ventilation were shorter in all groups when deceased patients were excluded, since patients who died during their hospital stay died relatively early in the postoperative phase. See results of unmatched patients in Supplementary Table S2. The results of the unmatched subgroup of patients undergoing elective CABG surgery and the subgroup of patients undergoing elective combined surgery or valve surgery are consistent with abovementioned results and show an even lower rate of adverse outcomes in the preop group when compared to the groups that received Levosimendan later. See Supplementary Tables S3–S6 for all morphometrical and outcome parameters of these subgroups.

**Table 2:** Outcomes of matched patients.

	[ALL]	Preop	Intraop	Postop	P. overall	*N*
	*N* = 234	*N* = 78	*N* = 78	*N* = 78		
In-hospital mortality	72 (30.8%)	13 (16.7%)	26 (33.3%)	33 (42.3%)	0.002	234
ICU mortality	72 (30.8%)	13 (16.7%)	26 (33.3%)	33 (42.3%)	0.002	234
LOS [d]	19.0 [10.0; 35.8]	18.0 [10.0; 28.8]	18.0 [11.0; 41.8]	24.0 [10.0; 42.8]	0.345	234
LOS [d] (excl. deceased)	23.5 [13.0; 39.0]	21.0 [11.0; 30.0]	19.5 [15.5; 43.0]	31.0 [16.0; 56.0]	0.003	162
ICU duration [d]	14.0 [8.00; 29.8]	13.5 [8.00; 26.0]	14.0 [8.00; 34.8]	16.0 [7.00; 36.8]	0.659	234
ICU duration [d] (excl. deceased)	17.5 [10.0; 34.0]	14.0 [9.00; 26.0]	17.0 [9.75; 36.5]	28.0 [13.0; 46.0]	0.009	162
Duration of mech. ventilation [h]	165 [76.0; 409]	112 [56.0; 286]	200 [84.0; 443]	204 [108; 552]	0.009	233
Duration of mech. ventilation [h] (excl. deceased)	165 [77.0; 411]	108 [54.0; 251]	200 [89.5; 472]	292 [125; 670]	0.001	161
CRRT	78 (33.3%)	18 (23.1%)	24 (30.8%)	36 (46.2%)	0.008	234

Outcomes of matched patients that received Levosimendan within the specified time frame. LOS, length of stay; CRRT, continuous renal replacement therapy w/o pre-existing renal insufficiency; Groups: preop, Levosimendan started at least one day before surgery, intraop, L. started on the day of surgery, postop, L. started one day after surgery or later.

## Discussion

Our study has several findings of note: postoperative Levosimendan administration following cardiac surgery to counteract already developed LCOS was associated with high mortality. Intraoperative Levosimendan treatment in patients with systolic left ventricular dysfunction, however, was associated with reduced mortality rates, compared to abovementioned postoperative administration. This finding is in line with previous studies, which analysed subgroups of large RCTs conducted in recent years (8, 11).

Most interestingly, prolonged preoperative treatment (“preconditioning”) of heterogeneous cardiac surgery patients preoperatively presenting with severely reduced ventricular function with Levosimendan was associated with reduced mortality when compared to patients who receive Levosimendan intraoperatively (16,7% vs. 33,3%). Also, duration of mechanical ventilation and incidence of CRRT were significantly lower in the preconditioning group. These associations were even stronger when deceased patients were excluded, who might otherwise introduce bias. This study thus extends recent knowledge in a large cohort of patients treated at a tertiary care hospital promoting preconditioning Levosimendan usage.

Previous research in the field has produced varying results concerning the value of perioperative Levosimendan, possibly because most RCTs have not taken full advantage of Levosimendan in their study protocols. To specify: as mentioned above, the unique pharmacokinetic profile of Levosimendan [see above and (14, 15)] recommends its administration well before the surgical/myocardial trauma occurs. In an uneventful intraoperative course, one might assume that commencing Levosimendan therapy during induction of anaesthesia and/or during weaning of CPB might be sufficient to at least “activate” cardioprotective cellular pathways. This may hold true especially if vulnerable myocardium has been verified preoperatively. Extending this hypothesis, one might expect an even more pronounced beneficial effect if most of the drug had been infused preoperatively. Such an association can be clearly seen in our data, including but not limited to elective CABG patients. This effect has been demonstrated before in a meta-analysis of two RCTs (11). Our findings are in line with the existing literature, which hints towards a positive effect of preconditioning patients with Levosimendan, but fails to find such an effect when Levosimendan is given only in the postoperative phase (8, 9, 13, 16). Additionally, a recent meta-analysis was able to demonstrate beneficial effects of Levosimendan in weaning patients from veno-arterial ECMO support (31), which is in line with our hypothesis that benefits of Levosimendan administration present themselves with a significant delay in onset.

To our knowledge, this retrospective study is the first to report a significant association of preconditioning and improved outcome not only in CABG patients, but in a cohort of patients undergoing all types of major cardiac surgery, i.e., CABG, valve surgery and combined surgery of CABG and valve (see Supplementary Tables S3–S6). We assume that our procedural change towards a more active screening of all cardiac surgery patients led to the abovementioned goal of reaching sufficient levels of Levosimendan or its metabolite before the surgery in high-risk patients. This is in line with the updated experts’ assessment (32) on the use of Levosimendan in the perioperative setting, based on two recent studies (33, 34). This experts’ assessment proposes the very early administration of Levosimendan in patients undergoing isolated CABG-surgery that exhibit severely reduced left ventricular function.

On the other hand, and not directly related to the administration of Levosimendan, the SOP change possibly increased alertness for a concomitant underlying ventricular dysfunction and pre-emptive therapeutic strategies. Previously, a patient’s ventricular (dys)function was in most cases known at admission, but might have been considered unamendable by preoperative optimisation. Rather, its recognition promoted some kind of “rescue strategy” intra- and/or postoperatively, e.g., administration of high-dose adrenergic catecholamines, Levosimendan, IABP and/or VAD placement. The SOP change towards preoperative screening of all patients might have improved the perioperative treatment of high-risk patients, synergistically with the preoperative administration of Levosimendan.

Of note, the maintenance of the SOP was internally discussed since the LEVO-CTS trial in 2017 (8) did not show a clear benefit of Levosimendan and the subgroup analysis in CABG patients with severely reduced left ventricular function had not been published yet (11). Thus, the implementation of preconditioning high-risk patients with Levosimendan was somewhat abandoned. In addition, the department of cardiac surgery was internally reorganized in 2018 being transferred towards a different location with new anaesthesia responsibilities. This might explain the decrease in Levosimendan treated patients in the preop group (Figure 1).

### Limitations

Our study is observational, and retrospective, and therefore has several important limitations.

• As mentioned before, the screening process itself in combination with heightened awareness of the treating physicians, especially the anaesthesia caregivers, might have played a significant role and introduced bias.
• We decided to include patients operated over a long time in our analysis, in order to increase the number of patients treated with Levosimendan, since after all only very few patients received this medication. While increasing the number of patients makes propensity score matching and statistical comparison in general more meaningful, it also means that (peri)procedural changes in our institution over time, other than the change of the SOP regarding Levosimendan administration, might have influenced patients’ outcome.
• We also cannot rule out that patients who received Levosimendan intra-/postoperatively, especially those after the SOP change, were patients that suffered from (peri)operative complications and are therefore not comparable to the patients who presented with preoperatively severely reduced ventricular function (“ultima ratio”).
• Additionally, we included operations that were classified as urgent/emergent and matched for this, but this might nevertheless introduce a bias. As group preop is defined as consisting of patients who received Levosimendan on the day before the surgery, this basically shifts all patients who have to be operated instantly, i.e., “high-grade” emergency, out of group preop. In addition, a selection towards patients who could tolerate at least 1 day of preconditioning which eventually might have leaded in postponing surgery could have taken place. See supplemental tables for subgroup analyses of patients who received elective surgeries only.
• Concerning data quality, we could not differentiate the precise origin and nature of valve dysfunctions leading to corrective surgery, e.g., mitral and/or tricuspid regurgitation.
• We did not have access to systematically recorded echocardiographic reports due to incompletely digitized patient records within the study period. Since the SOP of our institution required echocardiography by a (inter-)national certified echocardiographer before preconditioning patients with Levosimendan, we assume that all patients who were preconditioned with Levosimendan have thus shown severely reduced left ventricular systolic function according to the recent heart failure guideline (35).
• Additionally, our digital records did not include important surgical/perioperative variables that are known to have an impact on outcome, e.g., CPB time and cross-clamp time. Therefore, we could not use these additional variables in our propensity score matching, which might introduce significant bias.

## Conclusion

We have shown that establishing a screening process that aims to preoperatively identify cardiac surgical patients suffering from reduced left ventricular function and precondition these with Levosimendan is associated with significantly improved outcome when compared with patients who receive Levosimendan intra- or postoperatively. We speculate that this is predominantly caused by the pharmacodynamic properties of Levosimendan, but cannot rule out that the screening and preconditioning process, which initiates an “evaluation” period, itself in combination with experienced cardiac anaesthesia caregivers has played a part. Also, the information available to us in the form of digital records was lacking variables such as precise preoperative ejection fraction, CPB time and cross-clamp time, therefore our matching might have been suboptimal. We further speculate that this likely Levosimendan-induced effect has not widely been seen in previous randomized trials because these did not include prolonged preoperative administration of Levosimendan as an experimental stand-alone arm and/or administered the drug too broadly, i.e., previous studies did not limit its administration to patients suffering from severely reduced ventricular function strictly enough, and this might have masked its effect.

## Data Availability

The datasets presented in this article are not readily available because unaggregated data are not publicly available due to the possibility of de-anonymizing individual patients. Aggregated excerpts are available from the author FB (felix.balzer@charite.de) upon reasonable request. Requests to access the datasets should be directed to felix.balzer@charite.de.
