# Supplementary material for: Influence of timing of Levosimendan administration on outcomes in cardiac surgery
Source: Front Cardiovasc Med. 2023 Jul 26;10:1213696. doi: 10.3389/fcvm.2023.1213696 (PMC10410848; doi:10.3389/fcvm.2023.1213696)
Supplement: Supplementary file 1 [file Datasheet1.pdf]

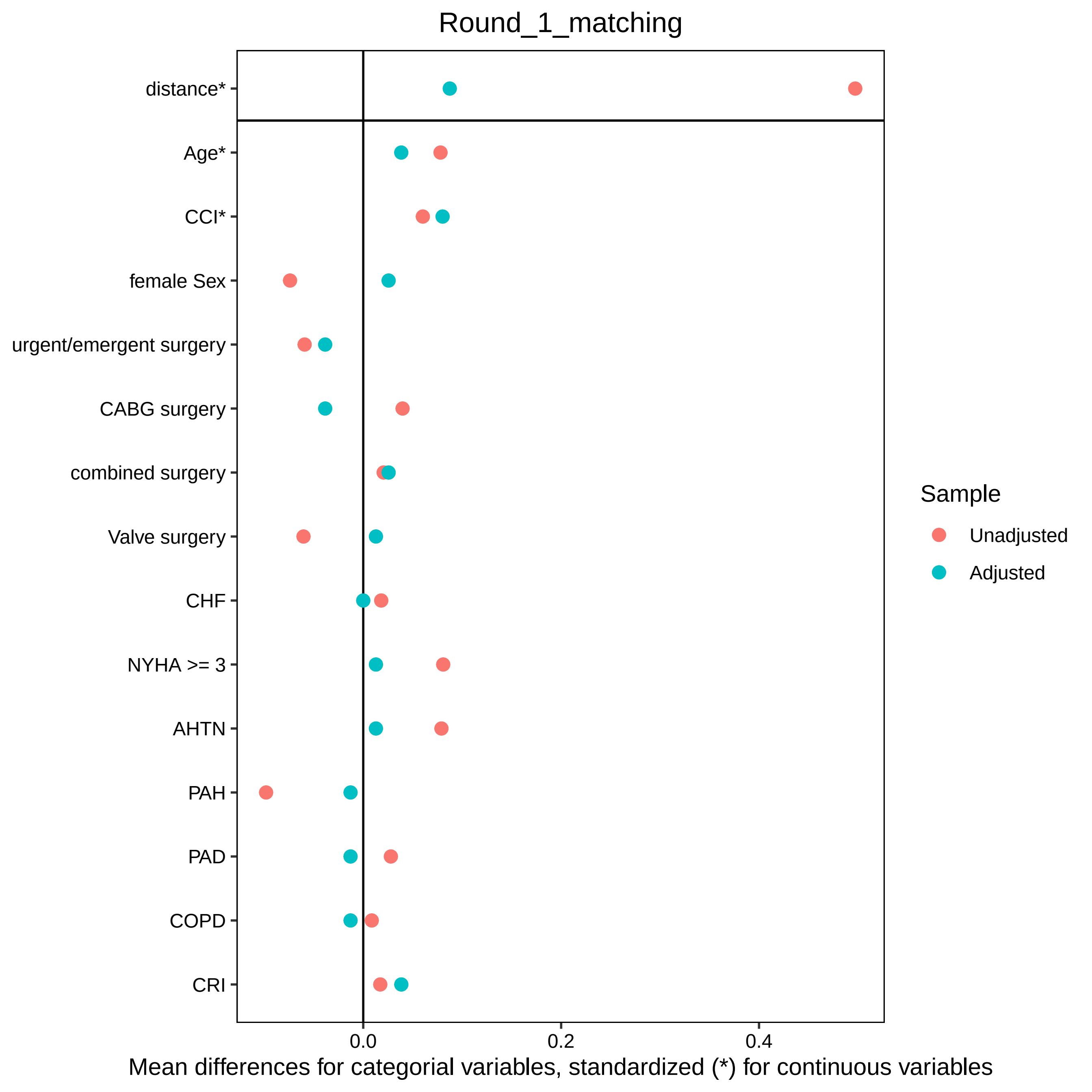

Round_1_matching
distance*
Age*
CCI*
female Sex
urgent/emergent surgery
CABG surgery
combined surgery
Valve surgery
CHF
NYHA >= 3
AHTN
PAH
PAD
COPD
CRI
0.0
0.2
0.4
Mean differences for categorial variables, standardized (*) for continuous variables
Sample
Unadjusted
Adjusted

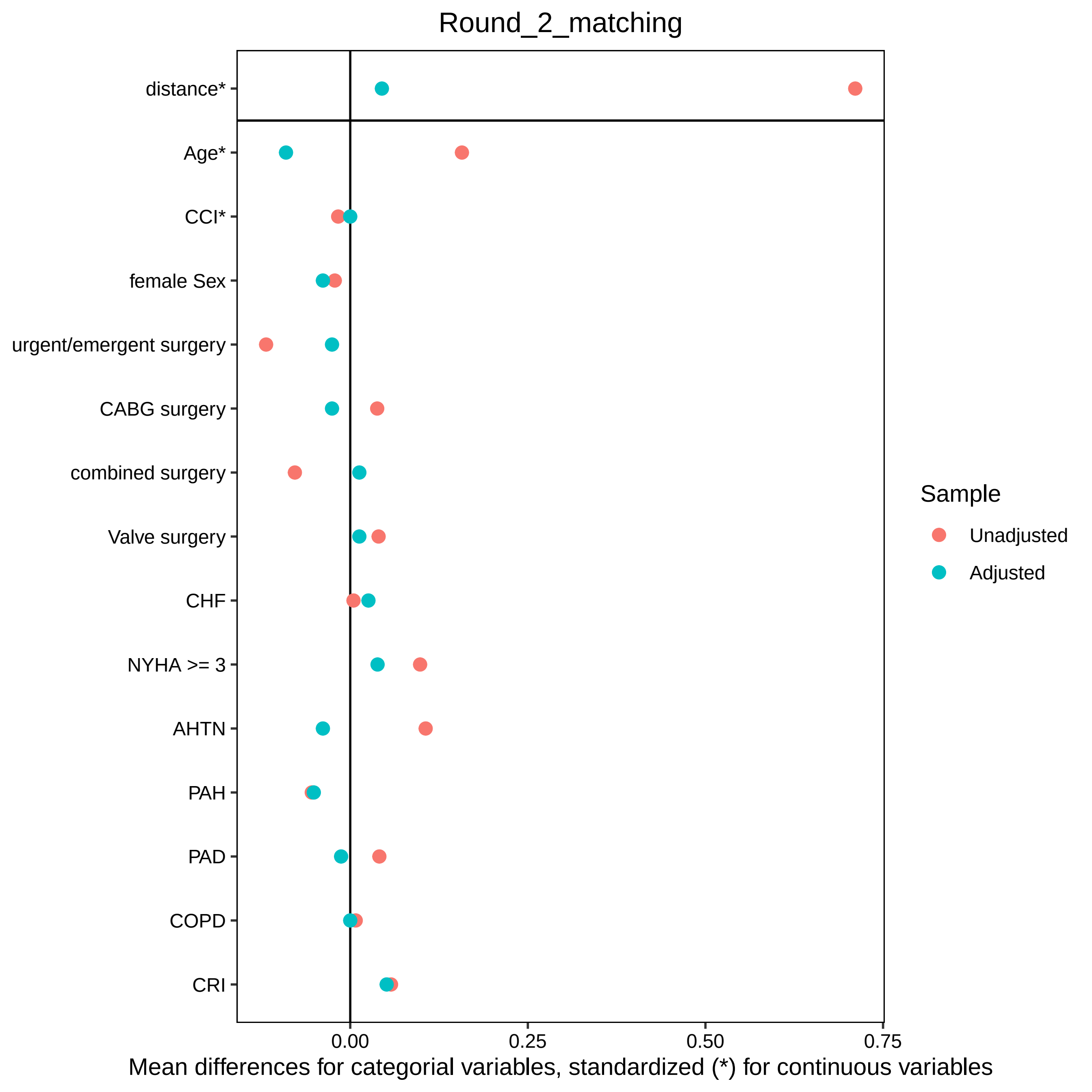
Round_2_matching
distance*
Age*
CCI*
female Sex
urgent/emergent surgery
CABG surgery
combined surgery
Valve surgery
CHF
NYHA >= 3
AHTN
PAH
PAD
COPD
CRI
0.00
0.25
0.50
0.75
Mean differences for categorial variables, standardized (*) for continuous variables
Sample
Unadjusted
Adjusted

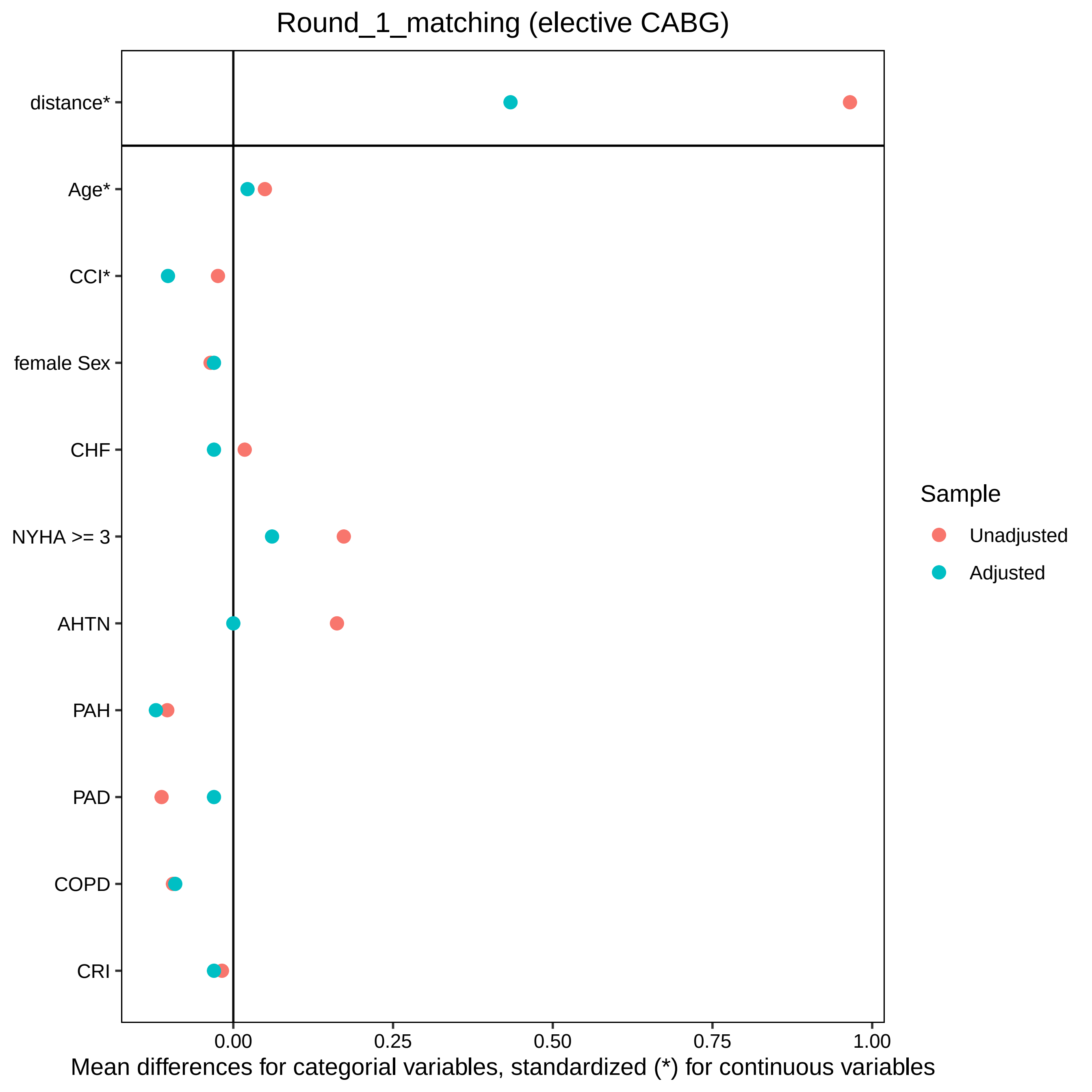
Round_1_matching (elective CABG)
distance*
Age*
CCI*
female Sex
CHF
NYHA >= 3
AHTN
PAH
PAD
COPD
CRI
0.00
0.25
0.50
0.75
1.00
Mean differences for categorial variables, standardized (*) for continuous variables
Sample
Unadjusted
Adjusted

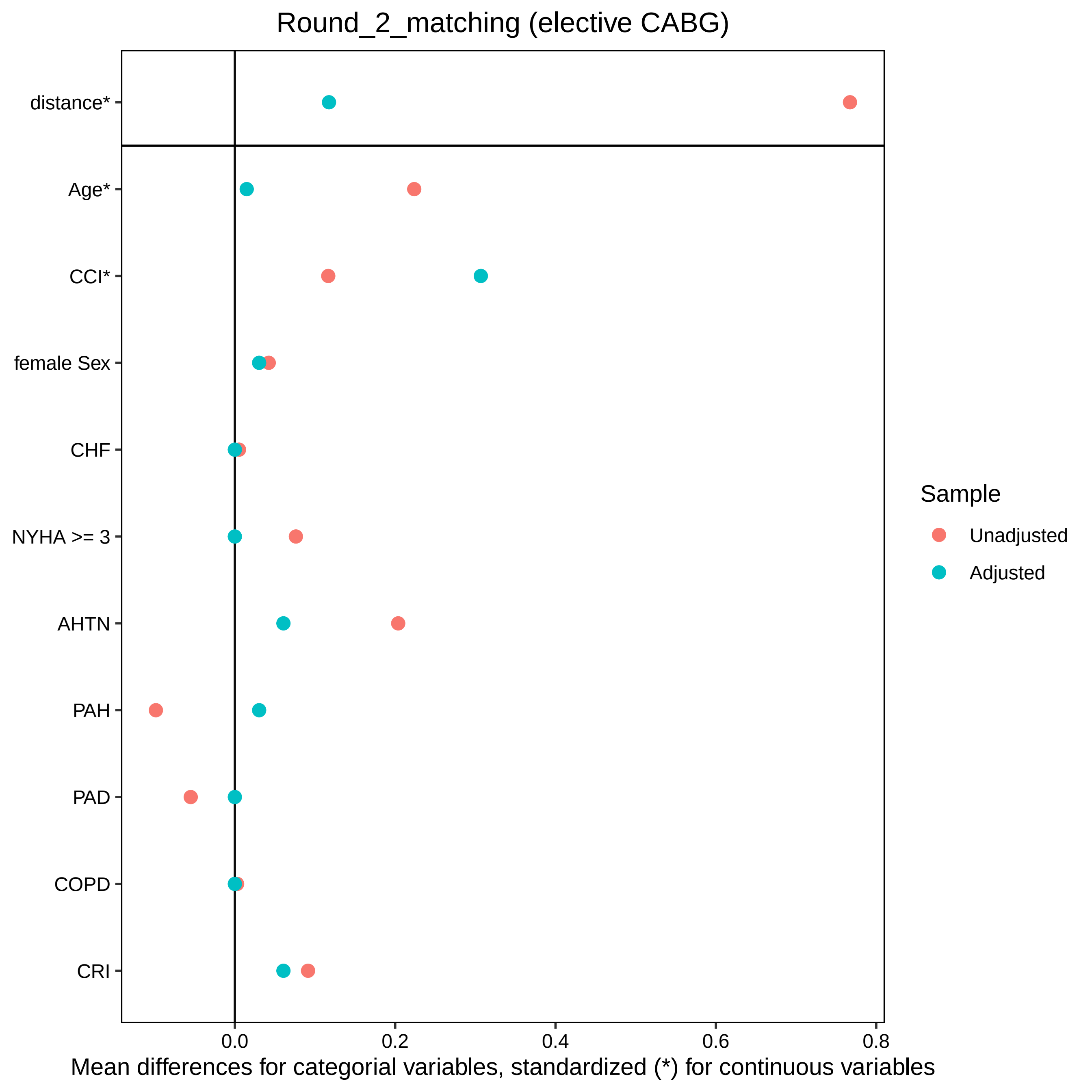
Round_2_matching (elective CABG)
distance*
Age*
CCI*
female Sex
CHF
NYHA >= 3
AHTN
PAH
PAD
COPD
CRI
0.0
0.2
0.4
0.6
0.8
Mean differences for categorial variables, standardized (*) for continuous variables
Sample
Unadjusted
Adjusted

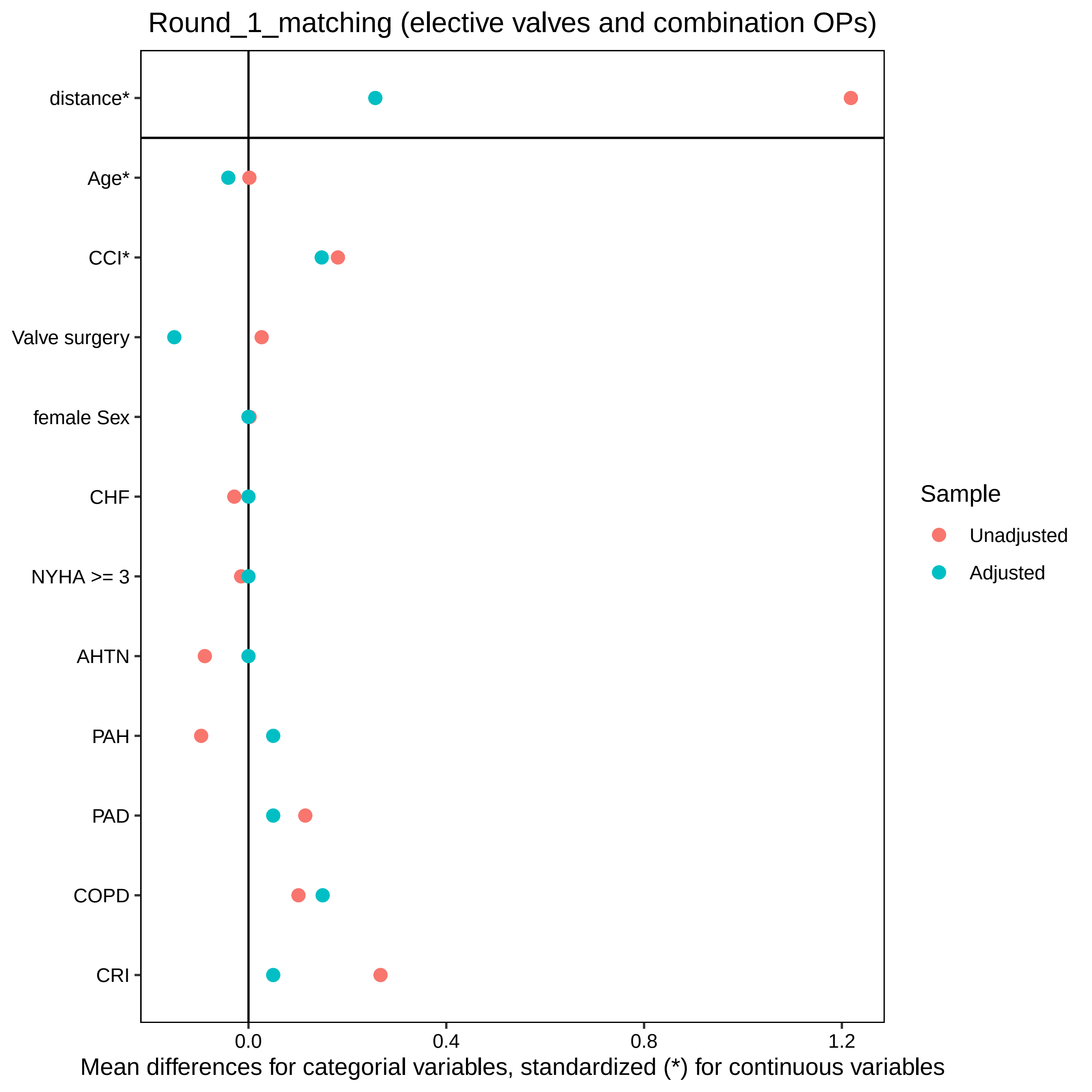
Round_1_matching (elective valves and combination OPs)
distance*
Age*
CCI*
Valve surgery
female Sex
CHF
NYHA >= 3
AHTN
PAH
PAD
COPD
CRI
0.0
0.4
0.8
1.2
Mean differences for categorial variables, standardized (*) for continuous variables
Sample
Unadjusted
Adjusted

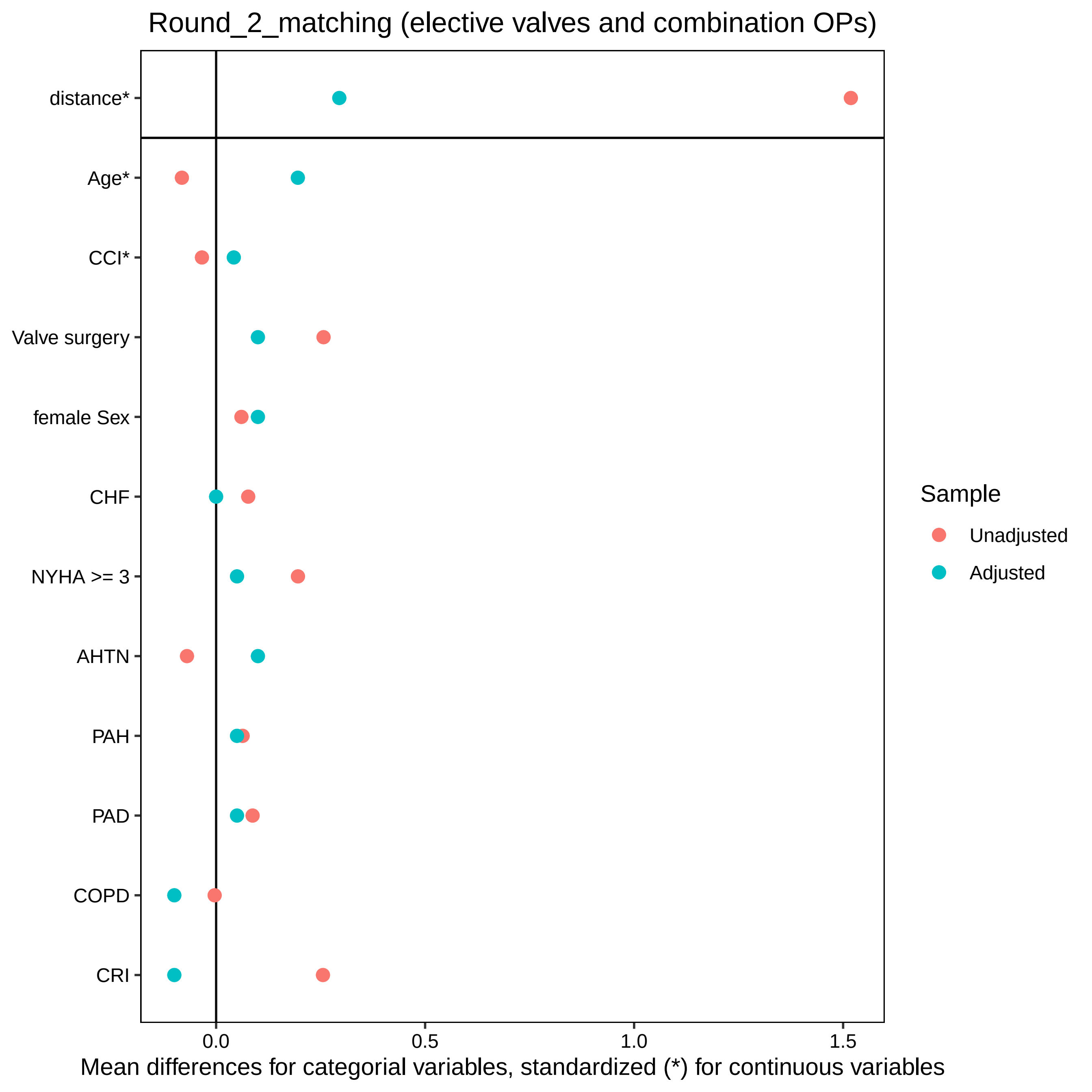

Round_2_matching (elective valves and combination OPs)
distance*
Age*
CCI*
Valve surgery
female Sex
CHF
NYHA >= 3
AHTN
PAH
PAD
COPD
CRI
0.0
0.5
1.0
1.5
Mean differences for categorial variables, standardized (*) for continuous variables
Sample
Unadjusted
Adjusted

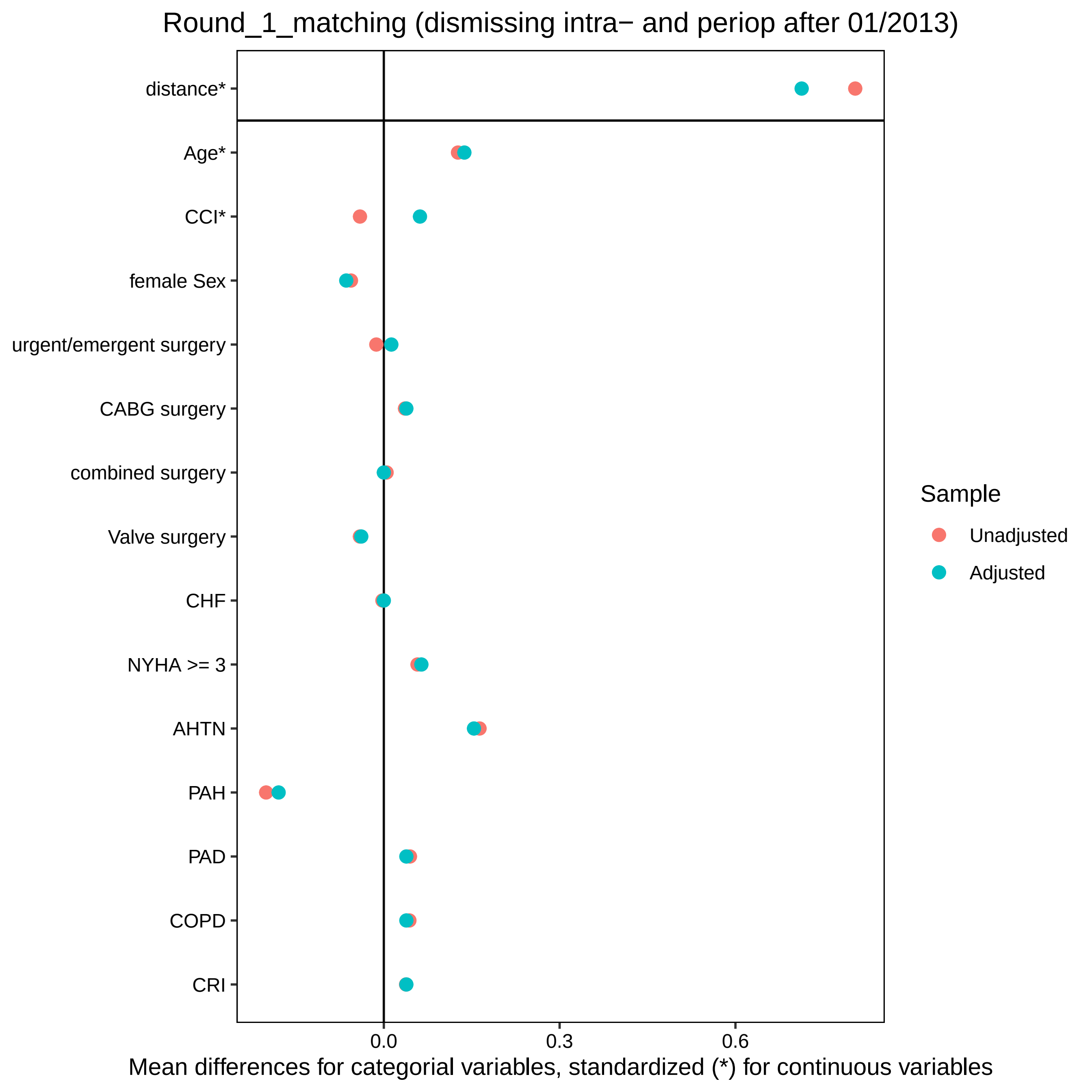
Round_1_matching (dismissing intra− and periop after 01/2013)
distance*
Age*
CCI*
female Sex
urgent/emergent surgery
CABG surgery
combined surgery
Valve surgery
CHF
NYHA >= 3
AHTN
PAH
PAD
COPD
CRI
0.0
0.3
0.6
Mean differences for categorial variables, standardized (*) for continuous variables
Sample
Unadjusted
Adjusted

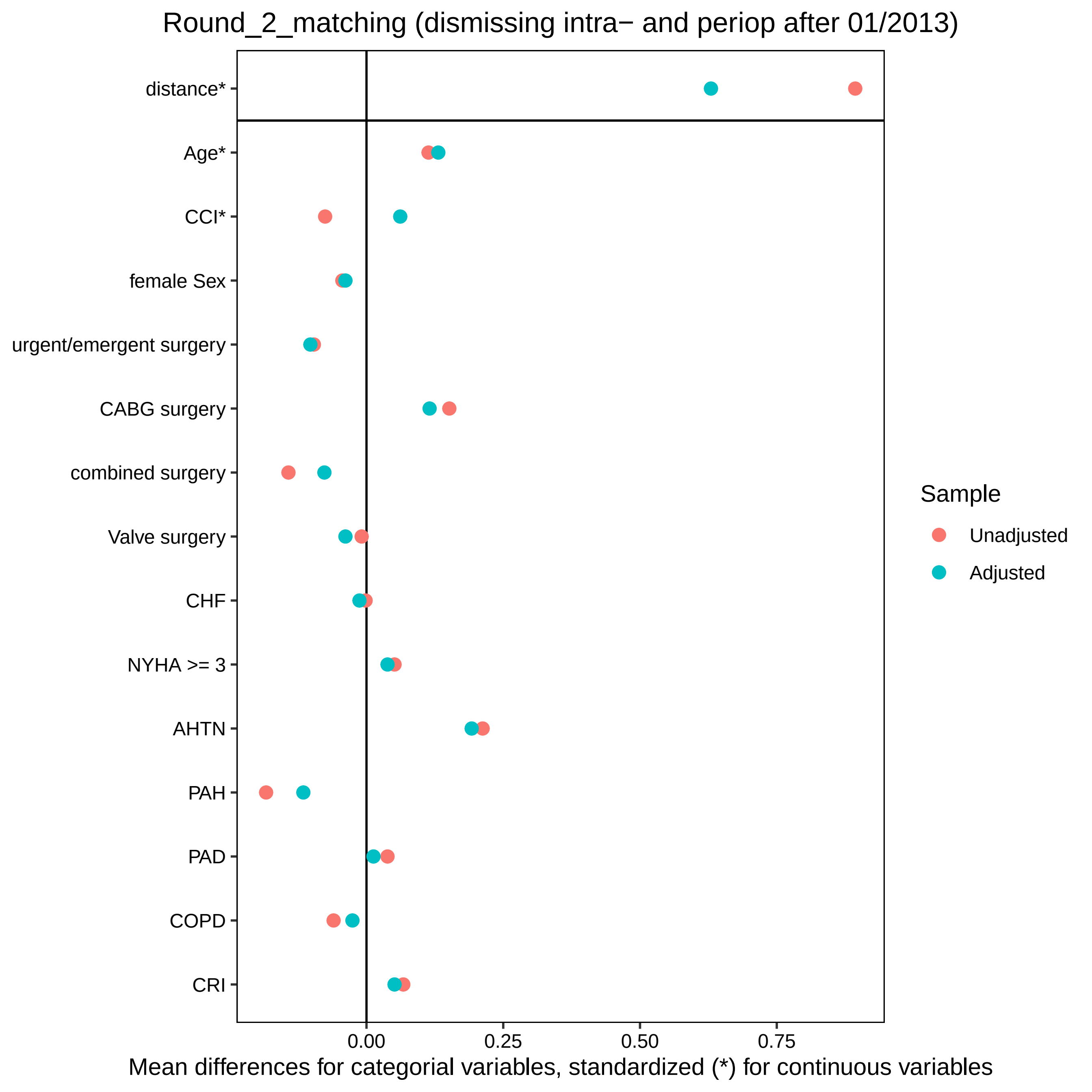

Round_2_matching (dismissing intra− and periop after 01/2013)
distance*
Age*
CCI*
female Sex
urgent/emergent surgery
CABG surgery
combined surgery
Valve surgery
CHF
NYHA >= 3
AHTN
PAH
PAD
COPD
CRI
0.00
0.25
0.50
0.75
Mean differences for categorial variables, standardized (*) for continuous variables
Sample
Unadjusted
Adjusted
