# Supplementary material for: Influence of timing of Levosimendan administration on outcomes in cardiac surgery
Source: Front Cardiovasc Med. 2023 Jul 26;10:1213696. doi: 10.3389/fcvm.2023.1213696 (PMC10410848; doi:10.3389/fcvm.2023.1213696)
Supplement: Supplementary file 2 [file Datasheet2.docx]

# Supplemental figure legends

**Supplemental figure 1**: distribution of delays between start of Levosimendan treatment and start of operation in matched patients (n = 234)

**Supplemental figure 2**: timing of Levosimendan administration in absolute numbers, umatched population (n = 498)

**Supplemental figure 3**: CONSORT flowchart of included patients and formation of groups

**Love plots:** Showing mean differences (of categorial variables) and standardized mean differences (of continuous variables) of every matching performed.
