# Supplementary material for: Influence of timing of Levosimendan administration on outcomes in cardiac surgery
Source: Front Cardiovasc Med. 2023 Jul 26;10:1213696. doi: 10.3389/fcvm.2023.1213696 (PMC10410848; doi:10.3389/fcvm.2023.1213696)
Supplement: Supplementary file 3 [file Datasheet3.docx]

**Supplemental table 1: baseline characteristics of unmatched patients**

|  | **[ALL]** | **preop** | **intraop** | **postop** | **p.overall** | **N** | **MD** |
| --- | --- | --- | --- | --- | --- | --- | --- |
|  | **N=498** | **N=78** | **N=262** | **N=158** |  |  |  |
| Age | 69.0 [60.0;76.0] | 71.0 [62.0;75.0] | 68.5 [60.0;75.0] | 69.0 [61.0;76.0] | 0.531 | 498 | 0.101 |
| Sex |  |  |  |  | 0.287 | 498 | 0.126 |
| M | 404 (81.1%) | 66 (84.6%) | 216 (82.4%) | 122 (77.2%) |  |  |  |
| W | 94 (18.9%) | 12 (15.4%) | 46 (17.6%) | 36 (22.8%) |  |  |  |
| BMI | 26.6 [23.8;30.1] | 27.6 [23.6;30.1] | 26.3 [23.4;30.2] | 26.5 [24.6;29.8] | 0.681 | 409 | 0.041 |
| Type of surgery |  |  |  |  | 0.026 | 498 | 0.228 |
| CABG | 303 (60.8%) | 50 (64.1%) | 158 (60.3%) | 95 (60.1%) |  |  |  |
| Combined | 81 (16.3%) | 10 (12.8%) | 54 (20.6%) | 17 (10.8%) |  |  |  |
| Valve | 114 (22.9%) | 18 (23.1%) | 50 (19.1%) | 46 (29.1%) |  |  |  |
| Urgency |  |  |  |  | 0.138 | 498 | 0.164 |
| elective | 298 (59.8%) | 53 (67.9%) | 147 (56.1%) | 98 (62.0%) |  |  |  |
| Urgent/Emergent | 200 (40.2%) | 25 (32.1%) | 115 (43.9%) | 60 (38.0%) |  |  |  |
| CCI | 6.00 [5.00;8.00] | 6.50 [5.00;8.00] | 6.00 [5.00;8.00] | 6.00 [4.25;8.00] | 0.733 | 498 | 0.046 |
| CHF | 462 (92.8%) | 73 (93.6%) | 244 (93.1%) | 145 (91.8%) | 0.834 | 498 | 0.047 |
| NYHA ≥ 3 | 402 (80.7%) | 69 (88.5%) | 206 (78.6%) | 127 (80.4%) | 0.153 | 498 | 0.178 |
| PAH | 151 (30.3%) | 19 (24.4%) | 78 (29.8%) | 54 (34.2%) | 0.292 | 498 | 0.145 |
| CAD | 430 (86.3%) | 66 (84.6%) | 232 (88.5%) | 132 (83.5%) | 0.312 | 498 | 0.097 |
| COPD | 86 (17.3%) | 14 (17.9%) | 45 (17.2%) | 27 (17.1%) | 0.985 | 498 | 0.015 |
| AHTN | 330 (66.3%) | 58 (74.4%) | 167 (63.7%) | 105 (66.5%) | 0.219 | 498 | 0.154 |
| PAD | 87 (17.5%) | 16 (20.5%) | 43 (16.4%) | 28 (17.7%) | 0.701 | 498 | 0.071 |
| Diabetes | 320 (64.3%) | 55 (70.5%) | 164 (62.6%) | 101 (63.9%) | 0.438 | 498 | 0.112 |
| CRI | 161 (32.3%) | 28 (35.9%) | 79 (30.2%) | 54 (34.2%) | 0.530 | 498 | 0.082 |

**Baseline characteristics of unmatched patients who received Levosimendan within the specified time frame.** Groups: preop = Levosimendan started at least one day before surgery, intraop = L. started on the day of surgery, postop = L. started one day after surgery or later. Groups: preop = Levosimendan started at least one day before surgery, intraop = L. started on the day of surgery, postop = L. started one day after surgery or later. CCI = Charlson Comorbidity Index, CHF = congestive heart failure, PAH = pulmonary arterial hypertension, CAD = coronary artery disease, COPD = chronic obstructive pulmonary disease, AHTN = arterial hypertension, PAD = peripheral arterial disease, CRI = chronic renal insufficiency. MD = mean differences, standardized mean differences for continuous variables (Age, BMI, CCI).

**Supplemental table 2: outcomes of unmatched patients**

|  | **[ALL]** | **preop** | **intraop** | **postop** | **p.overall** | **N** |
| --- | --- | --- | --- | --- | --- | --- |
|  | **N=498** | **N=78** | **N=262** | **N=158** |  |  |
| Intra-hospital mortality | 161 (32.3%) | 13 (16.7%) | 77 (29.4%) | 71 (44.9%) | <0.001 | 498 |
| ICU mortality | 160 (32.1%) | 13 (16.7%) | 76 (29.0%) | 71 (44.9%) | <0.001 | 498 |
| LOS [d] | 20.0 [11.0;35.8] | 18.0 [10.0;28.8] | 19.0 [11.0;34.8] | 21.0 [11.0;43.8] | 0.164 | 498 |
| LOS [d] (excl. deceased) | 24.0 [14.0;40.0] | 21.0 [11.0;30.0] | 22.0 [14.0;38.0] | 29.0 [16.5;56.0] | <0.001 | 337 |
| ICU duration [d] | 15.0 [8.00;29.0] | 13.5 [8.00;26.0] | 15.0 [8.00;28.0] | 17.0 [8.00;36.8] | 0.281 | 498 |
| ICU duration [d] (excl. deceased) | 18.0 [10.0;34.0] | 14.0 [9.00;26.0] | 17.0 [10.0;30.0] | 27.0 [14.0;44.5] | <0.001 | 337 |
| Duration of mech. ventilation [h] | 187 [76.0;471] | 112 [56.0;286] | 167 [62.0;402] | 250 [120;669] | <0.001 | 497 |
| Duration of mech. ventilation [h] (excl. deceased) | 171 [71.8;501] | 108 [54.0;251] | 152 [64.5;408] | 338 [158;702] | <0.001 | 336 |
| CRRT | 176 (35.3%) | 18 (23.1%) | 83 (31.7%) | 75 (47.5%) | <0.001 | 498 |

**Outcomes of unmatched patients that received Levosimendan within the specified time frame.** Groups: preop = Levosimendan started at least one day before surgery, intraop = L. started on the day of surgery, postop = L. started one day after surgery or later. LOS = length of stay, CRRT = continuous renal replacement therapy w/o pre-existing renal insufficiency

**Supplemental table 3: baseline characteristics of matched elective CABG patients**

|  | **[ALL]** | **preop** | **intraop** | **postop** | **p.overall** | **N** | **MD** |
| --- | --- | --- | --- | --- | --- | --- | --- |
|  | **N=99** | **N=33** | **N=33** | **N=33** |  |  |  |
| Age* | 68.3 (8.84) | 68.4 (8.19) | 68.3 (9.14) | 68.2 (9.42) | 0.996 | 99 | 0.014 |
| Sex* |  |  |  |  | 0.926 | 99 | 0.124 |
| M | 87 (87.9%) | 29 (87.9%) | 30 (90.9%) | 28 (84.8%) |  |  |  |
| W | 12 (12.1%) | 4 (12.1%) | 3 (9.09%) | 5 (15.2%) |  |  |  |
| BMI | 27.0 [24.6;30.1] | 27.8 [24.2;30.7] | 26.1 [24.5;28.4] | 26.7 [25.0;29.3] | 0.748 | 88 | 0.042 |
| Type of surgery: CABG | 99 (100%) | 33 (100%) | 33 (100%) | 33 (100%) | . | 99 |  |
| Urgency: elective | 99 (100%) | 33 (100%) | 33 (100%) | 33 (100%) | . | 99 |  |
| CCI* | 6.00 [5.00;8.00] | 7.00 [5.00;8.00] | 6.00 [5.00;7.00] | 7.00 [5.00;8.00] | 0.275 | 99 | 0.249 |
| CHF* | 94 (94.9%) | 31 (93.9%) | 31 (93.9%) | 32 (97.0%) | 1.000 | 99 | 0.097 |
| NYHA ≥ 3* | 85 (85.9%) | 29 (87.9%) | 29 (87.9%) | 27 (81.8%) | 0.818 | 99 | 0.113 |
| PAH* | 18 (18.2%) | 5 (15.2%) | 4 (12.1%) | 9 (27.3%) | 0.240 | 99 | 0.259 |
| CAD | 99 (100%) | 33 (100%) | 33 (100%) | 33 (100%) | . | 99 |  |
| COPD* | 15 (15.2%) | 4 (12.1%) | 4 (12.1%) | 7 (21.2%) | 0.493 | 99 | 0.164 |
| AHTN* | 82 (82.8%) | 28 (84.8%) | 26 (78.8%) | 28 (84.8%) | 0.753 | 99 | 0.105 |
| PAD* | 19 (19.2%) | 6 (18.2%) | 6 (18.2%) | 7 (21.2%) | 0.937 | 99 | 0.051 |
| Diabetes | 63 (63.6%) | 23 (69.7%) | 19 (57.6%) | 21 (63.6%) | 0.592 | 99 | 0.169 |
| CRI* | 38 (38.4%) | 13 (39.4%) | 11 (33.3%) | 14 (42.4%) | 0.742 | 99 | 0.125 |

**Baseline characteristics of matched elective CABG patients who received Levosimendan within the specified time frame.** Matched (*) on age + sex + Charlson Comorbidity Index (CCI) + congestive heart failure (CHF) + NYHA ≥ 3 + pulmonary hypertension (PAH) + chronic obstructive pulmonary disease (COPD) + arterial hypertension (AHTN) + peripheral arterial disease (PAD) and chronic renal insufficiency (CRI).Groups: preop = Levosimendan started at least one day before surgery, intraop = L. started on the day of surgery, postop = L. started one day after surgery or later. CAD = coronary artery disease. MD = mean differences, standardized mean differences for continuous variables (Age, BMI, CCI).

**Supplemental table 4: outcomes of matched elective CABG patients**

|  | **[ALL]** | **preop** | **intraop** | **postop** | **p.overall** | **N** |
| --- | --- | --- | --- | --- | --- | --- |
|  | **N=99** | **N=33** | **N=33** | **N=33** |  |  |
| Intra-hospital mortality | 22 (22.2%) | 4 (12.1%) | 6 (18.2%) | 12 (36.4%) | 0.048 | 99 |
| ICU mortality | 22 (22.2%) | 4 (12.1%) | 6 (18.2%) | 12 (36.4%) | 0.048 | 99 |
| LOS [d] | 20.0 [10.5;30.0] | 16.0 [11.0;27.0] | 15.0 [9.00;27.0] | 26.0 [13.0;42.0] | 0.067 | 99 |
| LOS [d] (excl. deceased) | 22.0 [11.0;32.0] | 19.0 [11.0;27.0] | 18.0 [9.50;27.5] | 30.0 [20.0;43.0] | 0.022 | 77 |
| ICU duration [d] | 14.0 [8.00;26.0] | 12.0 [9.00;25.0] | 10.0 [8.00;17.0] | 21.0 [10.0;37.0] | 0.114 | 99 |
| ICU duration [d] (excl. deceased) | 14.0 [9.00;26.0] | 14.0 [10.0;25.0] | 11.0 [8.00;18.0] | 26.0 [13.0;42.0] | 0.092 | 77 |
| Duration of mech. ventilation [h] | 115 [63.0;349] | 96.0 [57.0;155] | 86.0 [54.0;228] | 275 [124;535] | 0.002 | 99 |
| Duration of mech. ventilation [h] (excl. deceased) | 103 [58.0;286] | 96.0 [56.0;155] | 76.0 [53.5;206] | 275 [124;699] | 0.014 | 77 |
| CRRT | 18 (18.2%) | 2 (6.06%) | 5 (15.2%) | 11 (33.3%) | 0.014 | 99 |

**Outcomes of matched elective CABG patients who received Levosimendan within the specified time frame.** Groups: preop = Levosimendan started at least one day before surgery, intraop = L. started on the day of surgery, postop = L. started one day after surgery or later. LOS = length of stay, CRRT = continuous renal replacement therapy w/o pre-existing renal insufficiency

**Supplemental table 5: baseline characteristics of matched patients undergoing elective combined surgery and valve surgery**

|  | **[ALL]** | **preop** | **intraop** | **postop** | **p.overall** | **N** | **MD** |
| --- | --- | --- | --- | --- | --- | --- | --- |
|  | **N=60** | **N=20** | **N=20** | **N=20** |  |  |  |
| Age* | 70.5 [59.5;75.0] | 70.5 [62.0;74.0] | 63.5 [57.8;74.2] | 71.5 [58.0;76.0] | 0.673 | 60 | 0.151 |
| Sex* |  |  |  |  | 0.711 | 60 | 0.155 |
| M | 44 (73.3%) | 14 (70.0%) | 16 (80.0%) | 14 (70.0%) |  |  |  |
| W | 16 (26.7%) | 6 (30.0%) | 4 (20.0%) | 6 (30.0%) |  |  |  |
| BMI | 26.2 (4.75) | 26.6 (6.08) | 26.5 (3.84) | 25.6 (4.02) | 0.776 | 53 | 0.153 |
| Type of surgery* |  |  |  |  | 0.207 | 60 | 0.417 |
| Combined | 14 (23.3%) | 5 (25.0%) | 7 (35.0%) | 2 (10.0%) |  |  |  |
| Valve | 46 (76.7%) | 15 (75.0%) | 13 (65.0%) | 18 (90.0%) |  |  |  |
| Urgency: elective | 60 (100%) | 20 (100%) | 20 (100%) | 20 (100%) | . | 60 |  |
| CCI * | 6.15 (2.21) | 6.30 (2.36) | 6.20 (2.35) | 5.95 (1.99) | 0.879 | 60 | 0.106 |
| CHF * | 57 (95.0%) | 19 (95.0%) | 19 (95.0%) | 19 (95.0%) | 1.000 | 60 |  |
| NYHA ≥ 3* | 53 (88.3%) | 18 (90.0%) | 17 (85.0%) | 18 (90.0%) | 1.000 | 60 | 0.101 |
| PAH* | 28 (46.7%) | 10 (50.0%) | 9 (45.0%) | 9 (45.0%) | 0.935 | 60 | 0.067 |
| CAD | 34 (56.7%) | 10 (50.0%) | 16 (80.0%) | 8 (40.0%) | 0.029 | 60 | 0.586 |
| COPD* | 14 (23.3%) | 5 (25.0%) | 7 (35.0%) | 2 (10.0%) | 0.207 | 60 | 0.417 |
| AHTN* | 31 (51.7%) | 11 (55.0%) | 9 (45.0%) | 11 (55.0%) | 0.766 | 60 | 0.134 |
| PAD* | 10 (16.7%) | 4 (20.0%) | 3 (15.0%) | 3 (15.0%) | 1.000 | 60 | 0.088 |
| Diabetes | 32 (53.3%) | 14 (70.0%) | 10 (50.0%) | 8 (40.0%) | 0.153 | 60 | 0.417 |
| CRI* | 40 (66.7%) | 13 (65.0%) | 15 (75.0%) | 12 (60.0%) | 0.592 | 60 | 0.216 |

**Baseline characteristics of matched elective valve and combination surgery patients who received Levosimendan within the specified time frame.** Matched (*) on age + sex + type of surgery + Charlson Comorbidity Index (CCI) + congestive heart failure (CHF) + NYHA ≥ 3 + pulmonary hypertension (PAH) + chronic obstructive pulmonary disease (COPD) + arterial hypertension (AHTN) + peripheral arterial disease (PAD) and chronic renal insufficiency (CRI). Groups: preop = Levosimendan started at least one day before surgery, intraop = L. started on the day of surgery, postop = L. started one day after surgery or later. CAD = coronary artery disease. MD = mean differences, standardized mean differences for continuous variables (Age, BMI, CCI).

**Supplemental table 6: outcomes of matched patients undergoing elective combined surgery and valve surgery**

|  | **[ALL]** | **preop** | **intraop** | **postop** | **p.overall** | **N** |
| --- | --- | --- | --- | --- | --- | --- |
|  | ***N=60*** | ***N=20*** | ***N=20*** | ***N=20*** |  |  |
| Intra-hospital mortality | 16 (26.7%) | 1 (5.00%) | 6 (30.0%) | 9 (45.0%) | 0.015 | 60 |
| ICU mortality | 15 (25.0%) | 1 (5.00%) | 5 (25.0%) | 9 (45.0%) | 0.014 | 60 |
| LOS [d] | 26.0 [16.0;42.5] | 25.5 [16.5;34.5] | 23.0 [15.5;37.2] | 40.5 [23.2;69.8] | 0.085 | 60 |
| LOS [d] (excl. deceased) | 24.5 [17.8;39.0] | 24.0 [16.0;35.0] | 23.0 [16.5;24.8] | 56.0 [28.5;62.0] | 0.010 | 44 |
| ICU duration [d] | 22.0 [11.8;35.2] | 19.5 [12.8;27.2] | 15.5 [8.75;32.2] | 29.0 [20.0;52.2] | 0.062 | 60 |
| ICU duration [d] (excl. deceased) | 22.0 [12.8;34.2] | 19.0 [12.5;26.5] | 15.5 [8.25;23.0] | 43.0 [26.5;50.5] | 0.003 | 44 |
| Duration of mech. ventilation [h] | 221 [76.0;497] | 161 [84.2;251] | 150 [51.2;653] | 400 [205;758] | 0.028 | 60 |
| Duration of mech. ventilation [h] (excl. deceased) | 166 [75.0;328] | 158 [70.5;250] | 86.5 [45.8;256] | 310 [231;636] | 0.021 | 44 |
| CRRT | 11 (18.3%) | 3 (15.0%) | 1 (5.00%) | 7 (35.0%) | 0.060 | 60 |

**Outcomes of matched elective valve and combination surgery patients who received Levosimendan within the specified time frame.** Groups: preop = Levosimendan started at least one day before surgery, intraop = L. started on the day of surgery, postop = L. started one day after surgery or later. LOS = length of stay, CRRT = continuous renal replacement therapy w/o pre-existing renal insufficiency

**Supplemental table 7: baseline characteristics of matched patients. Excluding patients who received Levosimendan intra- or postoperatively after January 2013**

|  | **[ALL]** | **preop** | **intraop** | **postop** | **p.overall** | **N** | **MD** |
| --- | --- | --- | --- | --- | --- | --- | --- |
|  | **N=234** | **N=78** | **N=78** | **N=78** |  |  |  |
| Age* | 70.0 [60.0;76.0] | 71.0 [62.0;75.0] | 70.5 [60.5;75.0] | 69.0 [59.2;76.0] | 0.775 | 234 | 0.086 |
| Sex* |  |  |  |  | 0.588 | 234 | 0.110 |
| M | 190 (81.2%) | 66 (84.6%) | 63 (80.8%) | 61 (78.2%) |  |  |  |
| W | 44 (18.8%) | 12 (15.4%) | 15 (19.2%) | 17 (21.8%) |  |  |  |
| BMI | 26.9 (4.64) | 27.3 (5.16) | 26.5 (4.55) | 26.8 (4.12) | 0.589 | 200 | 0.112 |
| Type of surgery* |  |  |  |  | 0.523 | 234 | 0.187 |
| CABG | 138 (59.0%) | 50 (64.1%) | 41 (52.6%) | 47 (60.3%) |  |  |  |
| Combined | 36 (15.4%) | 10 (12.8%) | 16 (20.5%) | 10 (12.8%) |  |  |  |
| Valve | 60 (25.6%) | 18 (23.1%) | 21 (26.9%) | 21 (26.9%) |  |  |  |
| Urgency of surgery* |  |  |  |  | 0.254 | 234 | 0.161 |
| Elective | 152 (65.0%) | 53 (67.9%) | 45 (57.7%) | 54 (69.2%) |  |  |  |
| Urgent/emergent | 82 (35.0%) | 25 (32.1%) | 33 (42.3%) | 24 (30.8%) |  |  |  |
| CCI* | 6.00 [5.00;8.00] | 6.50 [5.00;8.00] | 6.00 [5.00;7.75] | 6.00 [4.25;7.75] | 0.805 | 234 | 0.039 |
| CHF* | 220 (94.0%) | 73 (93.6%) | 74 (94.9%) | 73 (93.6%) | 1.000 | 234 | 0.037 |
| NYHA ≥ 3* | 199 (85.0%) | 69 (88.5%) | 66 (84.6%) | 64 (82.1%) | 0.528 | 234 | 0.121 |
| PAH* | 80 (34.2%) | 19 (24.4%) | 28 (35.9%) | 33 (42.3%) | 0.057 | 234 | 0.258 |
| CAD | 198 (84.6%) | 66 (84.6%) | 65 (83.3%) | 67 (85.9%) | 0.906 | 234 | 0.047 |
| COPD* | 41 (17.5%) | 14 (17.9%) | 16 (20.5%) | 11 (14.1%) | 0.570 | 234 | 0.113 |
| AHTN* | 147 (62.8%) | 58 (74.4%) | 43 (55.1%) | 46 (59.0%) | 0.031 | 234 | 0.273 |
| PAD* | 44 (18.8%) | 16 (20.5%) | 15 (19.2%) | 13 (16.7%) | 0.822 | 234 | 0.066 |
| Diabetes | 148 (63.2%) | 55 (70.5%) | 41 (52.6%) | 52 (66.7%) | 0.050 | 234 | 0.250 |
| CRI* | 77 (32.9%) | 28 (35.9%) | 24 (30.8%) | 25 (32.1%) | 0.778 | 234 | 0.073 |

**Baseline characteristics of matched patients that received Levosimendan within the specified time frame****. Excluding patients who received Levosimendan intra- or postoperatively after January 2013.** Matched (*) on age + sex + type of surgery + surgical urgency + Charlson Comorbidity Index (CCI) + congestive heart failure (CHF) + NYHA ≥ 3 + pulmonary hypertension (PAH) + chronic obstructive pulmonary disease (COPD) + arterial hypertension (AHTN) + peripheral arterial disease (PAD) and chronic renal insufficiency (CRI). Groups: preop = Levosimendan started at least one day before surgery, intraop = L. started on the day of surgery, postop = L. started one day after surgery or later. CAD = coronary artery disease. MD = mean differences, standardized mean differences for continuous variables (Age, BMI, CCI).

**Supplemental table 8: outcomes of matched patients. Excluding patients who received Levosimendan intra- or postoperatively after January 2013**

|  | **[ALL]** | **preop** | **intraop** | **postop** | **p.overall** | **N** |
| --- | --- | --- | --- | --- | --- | --- |
|  | **N=234** | **N=78** | **N=78** | **N=78** |  |  |
| Intra-hospital mortality | 78 (33.3%) | 13 (16.7%) | 26 (33.3%) | 39 (50.0%) | <0.001 | 234 |
| ICU mortality | 78 (33.3%) | 13 (16.7%) | 26 (33.3%) | 39 (50.0%) | <0.001 | 234 |
| LOS [d] | 21.0 [11.0;36.0] | 18.0 [10.0;28.8] | 19.5 [13.0;39.0] | 26.0 [10.2;44.0] | 0.127 | 234 |
| LOS [d] (excl. deceased) | 25.0 [14.0;39.2] | 21.0 [11.0;30.0] | 23.5 [15.8;41.5] | 30.0 [25.0;53.0] | <0.001 | 156 |
| ICU duration [d] | 15.0 [8.00;29.8] | 13.5 [8.00;26.0] | 14.0 [8.00;32.2] | 20.5 [7.25;37.8] | 0.289 | 234 |
| ICU duration [d] (excl. deceased) | 19.0 [9.75;33.2] | 14.0 [9.00;26.0] | 15.5 [9.00;34.0] | 29.0 [18.0;44.5] | 0.003 | 156 |
| Duration of mech. ventilation [h] | 188 [66.2;546] | 112 [56.0;286] | 210 [56.5;574] | 304 [116;704] | <0.001 | 234 |
| Duration of mech. ventilation [h] (excl. deceased) | 188 [67.5;559] | 108 [54.0;251] | 224 [79.8;652] | 557 [212;872] | <0.001 | 156 |
| CRRT | 90 (38.5%) | 18 (23.1%) | 30 (38.5%) | 42 (53.8%) | <0.001 | 234 |

**Outcomes of matched patients who received Levosimendan within the specified time frame. Excluding patients who received Levosimendan intra- or postoperatively after January 2013.** Groups: preop = Levosimendan started at least one day before surgery, intraop = L. started on the day of surgery, postop = L. started one day after surgery or later. LOS = length of stay, CRRT = continuous renal replacement therapy w/o pre-existing renal insufficiency
