# Supplementary material for: Influence of timing of Levosimendan administration on outcomes in cardiac surgery
Source: Front Cardiovasc Med. 2023 Jul 26;10:1213696. doi: 10.3389/fcvm.2023.1213696 (PMC10410848; doi:10.3389/fcvm.2023.1213696)
Supplement: Supplementary file 4 [file Datasheet4.docx]

**Supplementary figure 3 (CONSORT diagram)**

## Patient selection

Patients undergoing major cardiac surgery 2006 - 2018 (n = 11198)

Excluded (n = 10700)

- did not receive Levosimendan (n=10662)
- did receive L. earlier than 36 h preoperatively or later than 120 h postoperatively (n = 38)

Patients undergoing major cardiac surgery who received perioperative Levosimendan within specified timeframe (n = 498)

## Formation of groups

First administration of Levosimendan

on the day before surgery (or earlier) = ***preop*** (n = 78)

on the day of the surgery
= ***intraop*** (n = 262)

on the day following surgery or later = ***postop*** (n = 158)

## Matching (on age + sex + type of surgery + urgency + CCI + congestive heart failure + NYHA ≥ 3 + pulmonary arterial hypertension + chronic obstructive pulmonary disease + arterial hypertension + peripheral arterial disease + chronic renal insufficiency)

***postop*** (n = 78)

***intraop*** (n = 78)

***preop*** (n = 78)
