# Supplementary figures and images for: Influence of timing of Levosimendan administration on outcomes in cardiac surgery
Source: Front Cardiovasc Med. 2023 Jul 26;10:1213696. doi: 10.3389/fcvm.2023.1213696 (PMC10410848; doi:10.3389/fcvm.2023.1213696)

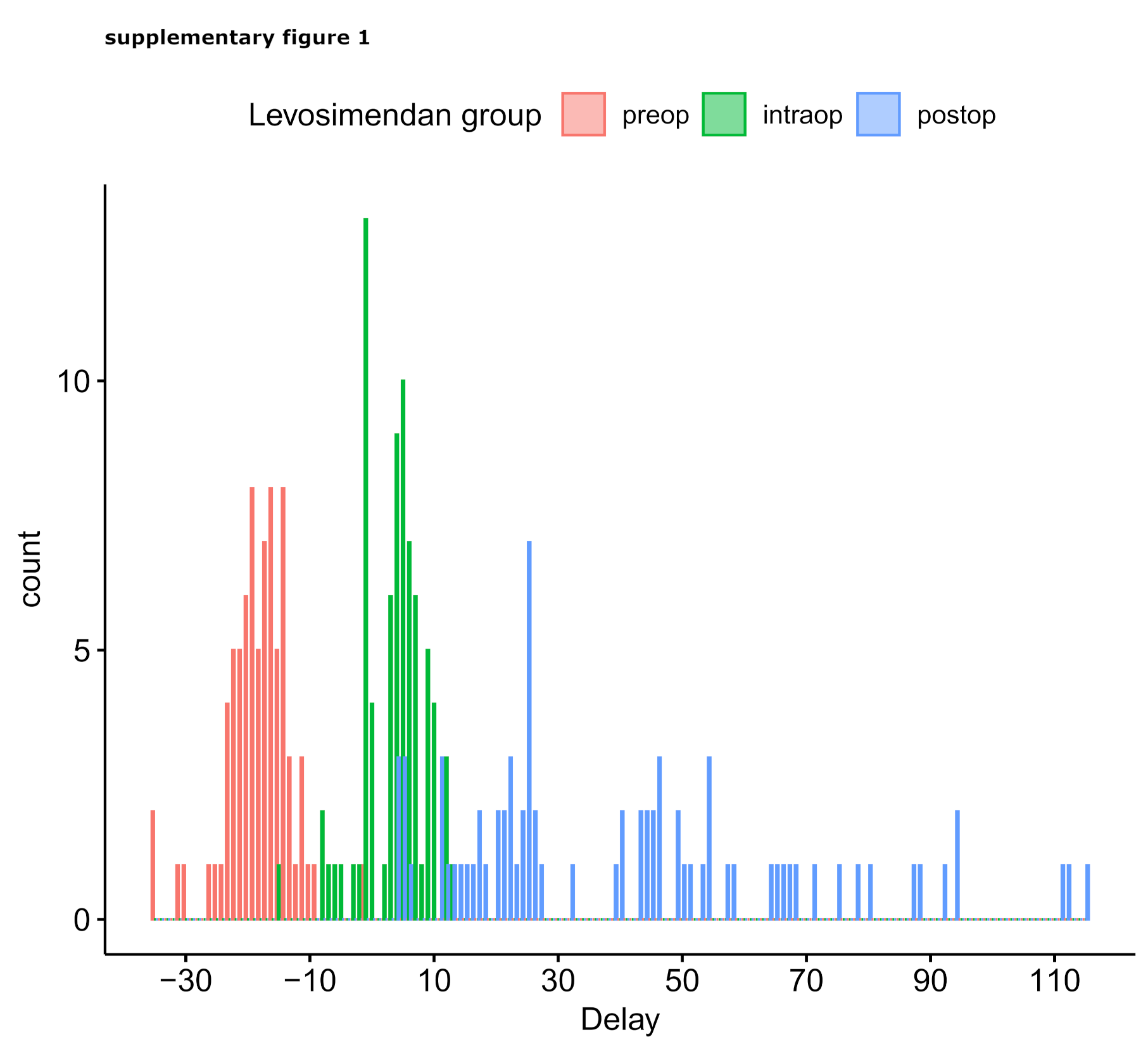

Supplement: Supplementary file 5 [file Image1.png]

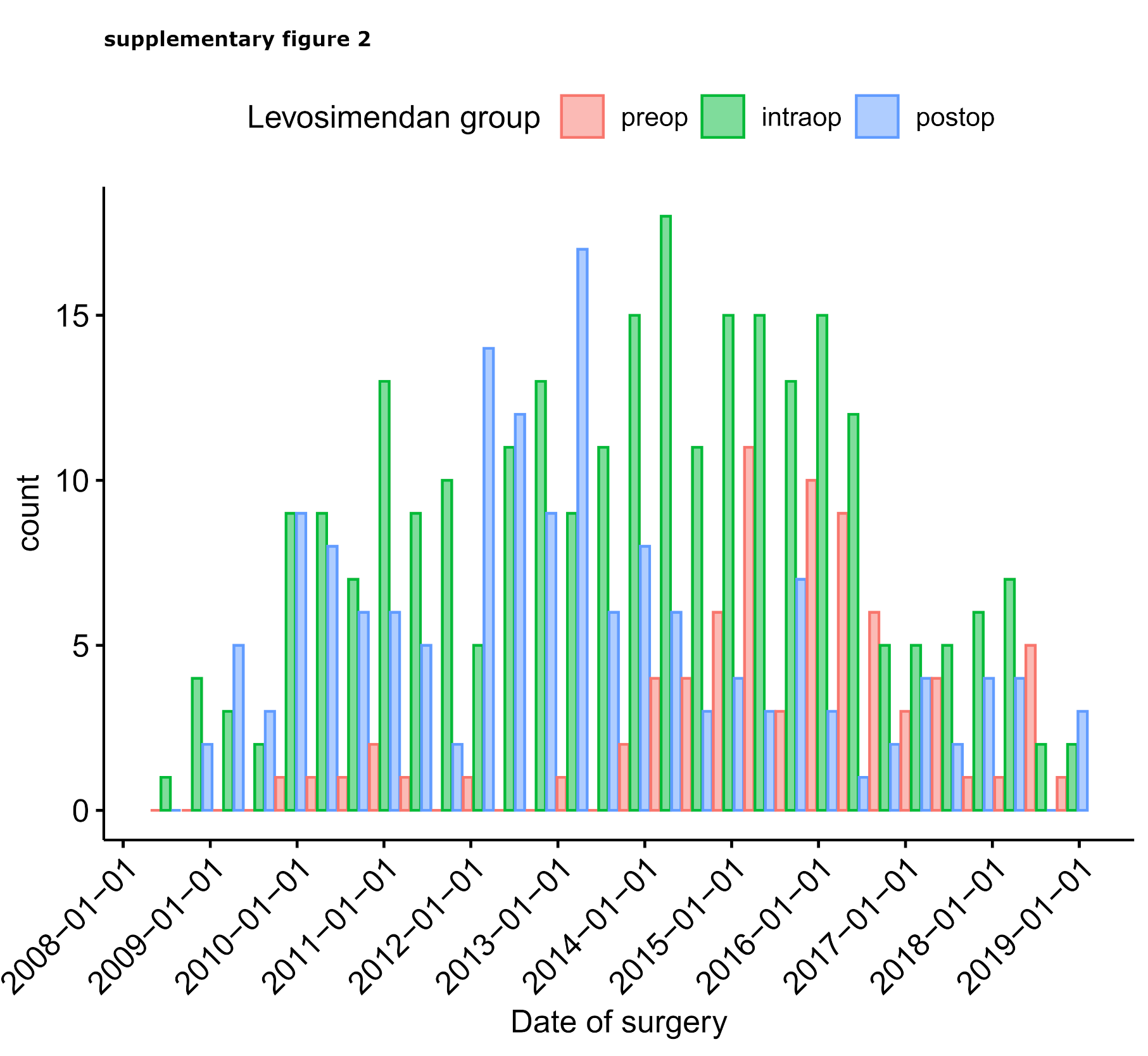

Supplement: Supplementary file 6 [file Image2.png]
